# Laryngeal lipoma: a rare cause of dysphonia

**DOI:** 10.11604/pamj.2017.26.9.10577

**Published:** 2017-01-04

**Authors:** Garrouche Nada, Jerbi Saida Omezzine, Dhifallah Maher, Ben Hamida Nouha, Hamza Hssine

**Affiliations:** 1 Radiology Department, Taher Sfar, Uuniversity Hospital Mahdia, Tunisia

**Keywords:** Laryngeal lipoma, dysphonia, mesenchymal tumors

## Abstract

Lipomas are the most common mesenchymal tumors. Laryngeal lipomas represent 1% of all lipomas but unlike other locations may cause life-threatening symptoms by obstruction of the respiratory tract. In this study, the case of a 32-year old woman with laryngeal lipoma is discussed. The lesion was detected on the left aryepiglottic fold, presented as a stalked and dynamic mass of 2 centimeters diameter. The imaging aspects of laryngeal lipoma cases, clinical evaluation, and approaches to treatment will be discussed.

## Introduction

Lipomas are the most common subcutaneous tumors. They may appear at any age, sex or body location. Lipomatous tumors in adults are frequent in the upper trunk, abdomen and shoulders. Rare in the first two decades, they manifest in the age where fat cells starts to accumulate in the body [1]. Their appearance in the head and neck is relatively uncommon, representing only 13% [1, 2]. Their location in the larynx represent only 1% [1, 3] and less than 115 cases have been reported in the literature [4]. The present case refers to a patient in whom a large pseudo-cystic mass, presenting in the left aryepiglottic fold, was revealed with following surgical removal. Upon histological examination, it was found to be a lipoma composed of mature adipocytes.

## Patient and observation

**Figure 1:**
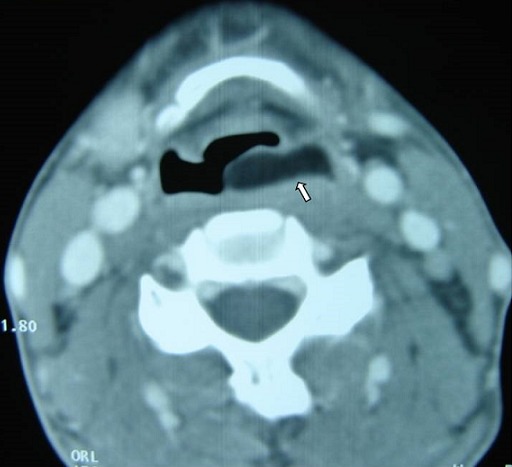
Axial CT scan of the neck revealed a well-defined mass of a very low-density without enhancement, highly suggestive of lipoma. This lesion, which was encapsulated, arose from the left para-laryngeal space (arrow)

**Figure 2:**
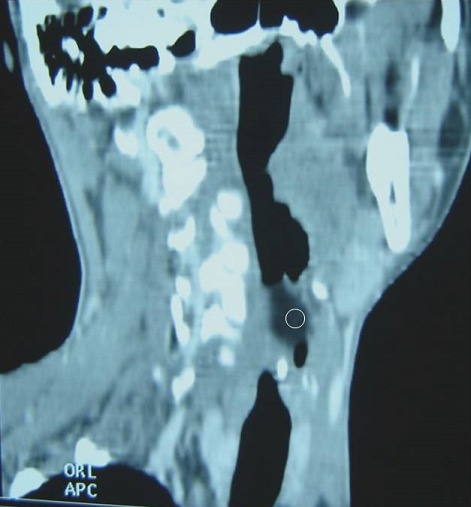
Sagittal reformation CT image: showed that this lesion (circle) presented an intra-luminal projecting portion that extended to the level of the hyoid bone

**Figure 3:**
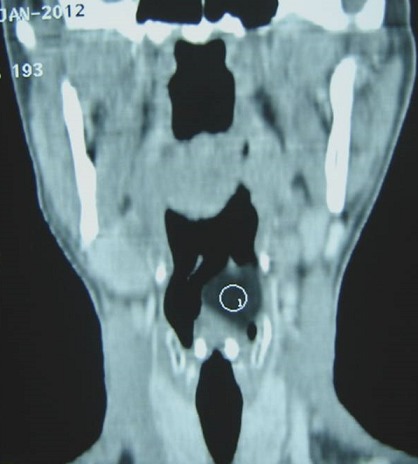
Coronal reformation CT image: showed that this lesion (circle) is exerting a compression on the pyriform sinus and the larynx, with respect to the neck vessels

A 32-year-old female patient came to our hospital complaining of changes in her voice, which had started several months earlier. She had not any complain of dysphagia or dyspnea. The physical examination was normal. Upon admission to ENT Department, the patient was submitted to Mirror examination of larynx which revealed a large submucosal swelling obliterating the left side of the supraglottic larynx and obscuring the airway. Mobility of left vocal cord was limited due to mass effect. A later Endoscopy showed the same findings: a large, smooth, pseudocystic mass, arising from the left aryepiglottic fold. This lesion was about 1.5 x 2 cm in size, of a translucent appearance and covered by normal, non-hemorrhagic mucosa. A computed tomography (CT) scan of the neck revealed a well-defined mass of a very low-density without enhancement, highly suggestive of lipoma (Figure 1). This lesion, which was encapsulated, arose from the left para-laryngeal space and presented an intra-luminal projecting portion that extended to the level of the hyoid bone (Figure 2), therefore exerting a compression on the pyriform sinus and the larynx, with respect to the neck vessels (Figure 3). Due to the site of this lesion, and the compression exerted on the surrounding anatomical structures, it was decided to proceed with surgical management, via an external (trans-cervical) approach, in order to ensure complete removal of the tumor. The Pathology examination revealed a 2cm encapsulated tumor. The mass was found to contain many uniform appearing mature adipocytes. As expected, macroscopic findings confirm the diagnosis of lipoma. The follow-up data revealed no evidence of recurrence.

## Discussion

The upper aero-digestive tract is a very rare location for Lipomas [5] which develop mostly in the posterior subcutaneous neck [6]. Lipomas are usually asymptomatic and gradually progressive in size which is the main reason for late diagnosis [7]. They mainly cause functional deficits like difficulty swallowing, neck pain and sleep apnea. The Diagnosis is difficult in this location and imaging methods are quite helpful to clinicians. Computerized tomography (CT) and magnetic resonance imaging (MRI) provide essential information for the management of these lesions [7–9]. CT scans help mainly in assessing the size and extent of the tumor. Laryngeal Lipomas appear frequently as pedunculated, single and straight-surfaced lesions [10]. On CT, adipose tissue is a non enhancing, homogenous low density areas (ranging within -64 to -123 Hounsfield Unit) [8, 9]. The differential diagnosis with malignant liposarcoma maybe difficult with the well differentiated form. MRI gives better tumor delineation as it has superior soft tissue contrast as well as clear definition of the location and extent of the mass [7]. It manifests a high signal intensity lesion on T1 weighted images and T2 weighted fast spin echo sequences.

These tumors do not metastasize; however, they have high rates of local recurrence and well-documented potential for delayed dedifferentiation into higher grade sarcomas(with potential for metastasis) [11]. The sarcomatoid degeneration on CT manifests as abnormal tumor margins and irregular vascularization [8, 11]. Simple lipomas, however, may also contain muscle fibers, blood vessels, fibrous septa, and areas of necrosis or inflammation. All these intra-lesional nonadipose components can confound the correct imaging diagnosis because they can mimic findings associated with well-differentiated liposarcoma [11]. Liposarcoma rarely rise from pre-existing lipomas and mostly arise denovo, but a few case of malignant change in lipomas have been described [11]. The true etiology of laryngeal lipoma is not clear. Multipotential fibroblast can differentiate into a fat cell through an unknown mechanism [12, 13]. The recent classification of benign lipomatous tumors includes the following categories: classic lipoma; lipoma variants, such as angiolipoma, chondroid lipoma, myolipoma and spindle cell/pleomorphic lipoma, all with specific clinical and histological features; hamartomatous lesions, diffuse lipomatous proliferations; and hibernoma [12, 14].

Lipomas usually present as solitary lesions, but multiple site involvement may be seen in alcoholics, diabetes mellitus and syndromes such as Madelung's disease and Kobberling-Dunningan syndrome [7]. Laryngeal lipomas may have extrinsic or intrinsic forms [10]. The intrinsic form of laryngeal lipomas is rare [15]. Within the 115 cases laryngeal lipomas reported in literature, only 30 are intrinsic [16]; this occurs in regions where Lipomatous tissues form a part of the subepithelial structures, such as in the false vocal cords, epiglottis, and aryepiglottic folds [16].

Laryngeal lipomas may be pedunculated or submucosal [17]. Pedunculated lipomas exert compression on adjacent anatomic structures and may cause airway obstruction. Submucosal lipomas deform the larynx and may cause partial airway obstruction and less phonatory disturbance. hoarseness seem to be the less common symptom [17].

The treatment of lipomas in head and neck is mainly by surgical excision in order to minimize the recurrence chance. Depending on size and extent of the tumor, it can be removed by endoscopic surgery or open surgery [17]. Recurrence may be indicative of low grade sarcoma and should be subjected to further investigation [18].

## Conclusion

Lipomas are rare ENT tumors. They cause few non specific symptoms and should be taken into consideration in the differential diagnosis of all benign head and neck masses.
